# Editorial: Promoting health and addressing disparities amongst Indigenous populations, volume II

**DOI:** 10.3389/fpubh.2026.1971595

**Published:** 2026-09-04

**Authors:** Jorge Vasconez-Gonzalez, Katherine A. Hirchak, Juan S. Izquierdo-Condoy, Esteban Ortiz-Prado

**Affiliations:** 1 One Health Research Group, Faculty of Health Sciences, Universidad de Las Américas, Quito, Ecuador; 2 Department of Community and Behavioral Health, Washington State University, Spokane, WA, United States

**Keywords:** health disparities, health inequalities, Indigenous, Indigenous knowledge, promoting health

## 1 Introduction

Indigenous Peoples are highly diverse, encompassing a wide range of cultures, languages, traditions, identities, and belief systems. Despite this diversity, many Indigenous Peoples have experienced historical and ongoing processes of exclusion and discrimination that have contributed to persistent health inequalities worldwide (1). These structural inequalities are reflected in shorter life expectancy, a higher burden of disease, a greater prevalence of risk factors for chronic conditions, and poorer quality of life (2). Moreover, many Indigenous knowledge systems conceptualize health holistically across the life course, encompassing physical and mental wellbeing as well as spiritual, cultural, and community wellbeing (3).

The Research Topic “*Promoting health and addressing disparities amongst Indigenous populations, volume II*” was conceived as a continuation of the first volume (4). It seeks to expand, deepen, and consolidate the body of knowledge concerning the health inequalities experienced by Indigenous Peoples worldwide, as well as the strategies and interventions being developed to address them. The strong engagement generated by the first volume, together with growing multidisciplinary interest and increasing research activity in this field, highlighted the value of developing a second Research Topic. Volume II builds on the previously published contributions by incorporating new evidence, perspectives, challenges, and experiences from diverse settings, thereby enriching our understanding of the opportunities and barriers involved in advancing health equity for Indigenous Peoples.

## 2 Contributions of volume II to the health of Indigenous Peoples

This volume brings together 16 articles addressing a broad range of topics and contexts relevant to understanding and responding to health inequalities among Indigenous Peoples. The contributions emphasize the importance of incorporating culturally appropriate and community-responsive approaches into the design, implementation, and evaluation of health policies and programs, while recognizing the diversity of Indigenous histories, priorities, needs, and realities. Collectively, they reaffirm the importance of collaborative research, community engagement, and equitable dialogue between Indigenous knowledge systems and academic or scientific approaches as foundations for developing more inclusive, sustainable, and effective responses to health inequalities.

### 2.1 Health promotion, health literacy, and innovation for prevention

The development of culturally appropriate, participatory, and community-responsive health-promotion strategies is a cornerstone of efforts to improve the health of Indigenous Peoples. Huang et al. reported a high prevalence of stunting and anemia among Tibetan infants aged 0–24 months in the context of traditional infant-feeding practices. Based on their findings, the authors proposed a conceptual framework integrating traditional feeding practices with evidence-based nutritional science. Magnusson et al. used mixed methods to evaluate the implementation of a brief, movement-based health-promotion workshop for healthcare professionals serving Indigenous communities in the Ecuadorian Highlands.

Several contributions also addressed health literacy and culturally appropriate health education. Liang et al. reported low levels of health literacy and moderate health outcomes among farmers living in the border regions of southern China. Welch et al. examined baseline knowledge and the perceived effects of an educational intervention addressing tick-borne diseases among Indigenous workers. Finally, Han et al. found that a human–artificial intelligence collaborative approach, combining AI-assisted structural optimization with expert review, could support the development of culturally appropriate educational materials for Chinese patients with temporomandibular disorders.

### 2.2 Disease prevention and management through community-based and culturally appropriate approaches

Both non-communicable and infectious diseases continue to pose substantial health challenges for Indigenous Peoples. Yang et al. assessed knowledge, attitudes, and practices regarding fatty liver disease among a community sample in Shanghai, China. Dubois et al. evaluated the effects of the COVID-19 pandemic on chronic disease management and on access to and use of healthcare services among Indigenous Peoples in Canada. Comiford et al. described the outcomes of a community-based Tribal program designed to identify and connect underserved individuals with screening, confirmatory testing, care, and treatment for hepatitis C, HIV, and syphilis within the Cherokee Nation in Oklahoma. Carrasco Rituay et al. analyzed HIV prevention intentions among Awajún and Wampis Indigenous communities in the Peruvian Amazon. Their findings revealed that long-term orientation and masculinity were positively and significantly associated with attitudes toward HIV prevention. Jansen et al. investigated concurrent alcohol and cigarette use among American Indian and Alaska Native adults enrolled in a randomized controlled trial of a contingency-management intervention for alcohol use disorder.

### 2.3 Culture, equity, and the transformation of health systems

Culture, self-determination, and equity-oriented health-system transformation are central to promoting wellbeing and reducing the health inequalities experienced by Indigenous Peoples. Hirchak et al. established culturally grounded conceptual foundations for the development and implementation of a clinical trial addressing alcohol use among Indigenous emerging adults. Similarly, Ka'ula et al. examined the cultivation of kalo, or taro, as a strategy for strengthening culture and promoting health. Their findings suggest that community gardens dedicated to traditional foods can support both health and cultural identity within displaced Indigenous communities.

Veenstra et al. developed an evidence-informed and equity-oriented readiness-assessment tool adapted to the Aotearoa New Zealand context and intended to support the implementation of health interventions in Māori communities. Abdelmalek and Farrugia examined denialism and its role in perpetuating anti-Indigenous racism in healthcare in Canada, highlighting its implications for health equity and the transformation of health systems.

### 2.4 Capacity building, data governance, and innovation for Indigenous research

Strengthening Indigenous research capacity requires sustained investment in education, data governance, ethical frameworks, and institutional practices that respect Indigenous sovereignty. Smith et al. described a collaborative initiative between a university in North Dakota and a Tribal college to develop a summer data-science program for students attending Tribal colleges in the state. Similarly, Beans et al. emphasized the responsibility of both Indigenous and non-Indigenous researchers to recognize Tribal sovereignty and adhere to appropriate ethical principles, regulations, and community-governance requirements when conducting genetic research and collecting data involving Indigenous Peoples.

## 3 Conclusion

The 16 articles included in this Research Topic illustrate the complexity of the challenges and structural barriers affecting the health of Indigenous Peoples while also presenting strategies that may contribute to advancing health equity. Collectively, these contributions underscore the importance of developing culturally appropriate and community-responsive interventions, strengthening health literacy, promoting the meaningful participation of Indigenous communities, and transforming health systems to make them more inclusive, equitable, and culturally safe.

Future research and policy initiatives should move beyond documenting disparities and actively support Indigenous leadership, sovereignty, and participation in the production and governance of research and health knowledge. Sustained partnerships among Indigenous communities, researchers, health professionals, and policymakers will be essential for developing equitable responses that reflect the distinct histories, priorities, and realities of Indigenous Peoples that are necessary for improved and lasting health outcomes.
